# Beyond Chronological Age: Frailty and Multimorbidity Predict In-Hospital Mortality in Patients With COVID-19

**DOI:** 10.1093/geroni/igab046.055

**Published:** 2021-12-17

**Authors:** Alberto Zucchelli, Alessandra Marengoni, Davide Vetrano, Luigi Ferrucci, Laura Fratiglioni, Roberto Bernabei, Graziano Onder, Giuseppe Romanelli

**Affiliations:** 1 Università degli Studi di Brescia, Karolinska Institutet, Stockholms Lan, Sweden; 2 University of Brescia, Brescia, Lombardia, Italy; 3 Karolinska Institutet, Solna, Stockholms Lan, Sweden; 4 National Institute on Aging, Baltimore, Maryland, United States; 5 Karolinska Institutet 17175, Stockholms Lan, Sweden; 6 Catholic University of Rome, Rome, Lazio, Italy; 7 Italian National Institute of Health, Rome, Lazio, Italy; 8 Università degli Studi di Brescia, Università degli Studi di Brescia, Lombardia, Italy

## Abstract

Background: We evaluated whether frailty and multimorbidity predict in-hospital mortality in patients with COVID-19 beyond chronological age. Methods: 165 patients admitted from March 8th to April 17th, 2020, with COVID-19 in an acute geriatric ward in Italy were included. Pre-disease frailty was assessed with the Clinical Frailty Scale (CFS). Multimorbidity was defined as the co-occurrence of ≥2 of these in the same patient. The hazard (HR) of in-hospital mortality as a function of CFS score and number of chronic diseases in the whole population and in those aged 70+ years were calculated. Results Among the 165 patients, 112 were discharged, 11 were transferred to intensive care units and 42 died. Patients who died were older (81.0 vs. 65.2 years, p<0.001), more frequently multimorbid (97.6 vs. 52.8%; p<0.001) and more likely frail (37.5 vs. 4.1%; p<0.001). Less than 2.0% of patients without multimorbidity and frailty, 28% of those with multimorbidity only and 75% of those with both multimorbidity and frailty died. Each unitary increment in the CFS was associated with a higher risk of in-hospital death in the whole sample (HR=1.3; 95%CI=1.05-1.62) and in patients aged 70+ years (HR=1.29;95%CI=1.04-1.62), whereas the number of chronic diseases was not significantly associated with higher risk of death. The CFS addition to age and sex increased mortality prediction by 9.4% in those aged 70+ years. Conclusions Frailty identifies patients with COVID-19 at risk of in-hospital death independently of age. Multimorbidity contributes to prognosis because of the very low probability of death in its absence.

